# Uptake of liquid-based cytology as an adjunct to conventional cytology for cervical screening in NSW, Australia: a cross-sectional and population-based cohort analysis

**DOI:** 10.1186/1471-2458-13-1196

**Published:** 2013-12-18

**Authors:** Nayyereh Aminisani, Bruce K Armstrong, Karen Canfell

**Affiliations:** 1 (Current affiliation) Tabriz University of Medical Sciences, Tabriz, Iran; 2 Sydney School of Public Heath, University of Sydney, City Road, Camperdown, NSW, Australia; 3 (Current affiliation) Lowy Cancer Research Centre, Prince of Wales Clinical School, The University of NSW, Sydney, Australia; 4 (Past affiliations) Cancer Research Division, Cancer Council NSW, Woolloomooloo, Australia

**Keywords:** Cervical screening, Cervical cytology, Pap smear, Liquid based cytology, Australia

## Abstract

**Background:**

Cervical screening is currently recommended every two years in sexually active women aged 18-20 to 69 years in Australia. Direct replacement of conventional cytology with liquid-based cytology (LBC) for cervical screening was rejected for public funding on grounds of cost-effectiveness, first in 2002 and again in 2009, but LBC is performed as an adjunct to conventional cytology in women who elect to pay. The objective of this study was to describe prevalence and predictors of use of LBC in Australia’s most populous state, New South Wales (NSW).

**Methods:**

We performed cross-sectional and population-based cohort analyses using data from the state Pap Test Register in NSW. We calculated the age-adjusted proportion of women aged 20-69 years electing to have adjunctive LBC over the period from 2006-2010. We also calculated the fully-adjusted odds ratios for the association between subsequent LBC use and age, socioeconomic status, place of residence, previous cytological history and provider type in a cohort of 360,247 women who had an index cervical cytology test in 2006–8.

**Results:**

Uptake of LBC varied between 29.7% (95% Confidence Interval (CI): 29.5-30.0%) in 2006/7 and 26.6% (95% CI: 26.4-26.9%) in 2009/10. LBC was more likely to be used in women aged 30-44 years, if it had been used previously (OR13.58, 95% CI: 13.33-13.84), if the previous test result was abnormal (OR2.62, 95% CI:2.53-2.72) or unsatisfactory (OR2.37, 95% CI:2.27-3.47), or if a gynaecologist requested the test (OR1.50, 95% CI:1.46-1.54). Uptake was least for women in remote/very remote areas (OR0.68; 95% CI:0.57-0.80 referenced to those in major cities) and in lower socioeconomic groups (OR 0.41, 95% CI:0.40-0.42 for lowest versus highest SES quintile).

**Conclusion:**

In the current environment in NSW, Australia, in which public funding for LBC has not been available, adjunctive uptake of LBC depends strongly on a woman’s age, her screening history and socioeconomic factors. These findings provide important context for a current review of technologies used in the National Cervical Screening Program in Australia.

## Background

The National Cervical Screening Program in Australia currently recommends that women are screened every 2 years between 18–20 and 69 years using conventional cytology [1]. However, organised cervical screening programs in several other countries, including England, Scotland and New Zealand, have replaced conventional cytology with liquid-based cytology (LBC), which is also now used for most cervical cytology tests in the USA. LBC is associated with fewer technically unsatisfactory tests, and hence fewer repeat tests, than conventional cytology, and this has been one of the underlying drivers of its cost-effectiveness in some settings, such as England, in which the unsatisfactory rate associated with conventional cytology was high (>7.5%) [2]. However, systematic reviews have found that manually-read LBC has close to equivalent sensitivity, but somewhat lower specificity, than conventional cytology for detection of biopsy-confirmed high grade precancerous cervical intraepithelial neoplasia (CIN2+) [3,4]. The evidence on the relative performance of image-read LBC (in which an automated computer imaging system is used to assist in slide reading) is much more limited, but two Australian studies have provided evidence to suggest that one of the available systems has an increased rate of detection of CIN2+ compared to conventional cytology [5,6]. The clarity of microscopic interpretation, improvements in laboratory processing, and potential for performing additional tests on the sample, including testing for the human papillomavirus (HPV), probably underpins the popularity of LBC with clinicians and pathologists [7]. In general terms, the ability to perform HPV DNA testing from the LBC sample is of increasing importance as HPV testing is considered for incorporation into screening programs, for example as a triage test for low grade cytological results. HPV DNA triage testing in conjunction with LBC screening is now used, for example, in England and New Zealand, and several other countries.

LBC has been evaluated by Australia’s Medical Services Advisory Committee (MSAC) as a direct replacement to conventional cytology on two previous occasions, and on both of these was rejected for public funding. A 2002 review concluded “there is currently insufficient evidence pertaining to liquid based cytology for cervical screening” [8], whereas a second review in 2009, which separately considered manually and image-read LBC, led MSAC to conclude that, “in comparison to the Papanicolaou (Pap) test, LBC is safe, is at least as effective, but is not cost effective at the price requested” [4], The cost-effectiveness finding was made in context of favourable assumptions about the relative sensitivity of LBC and was related, in part, to the low unsatisfactory rate currently experienced with conventional cytology in Australia (~2.2%) [4,9], and to the shorter recommended screening interval compared to that in other countries [4,10]. Currently, LBC is available as an adjunctive test if women elect to pay, with the price varying according to laboratory (up to ~ $40-$60). Adjunctive testing is performed using the split-sample technique, in which a conventional cytology slide is prepared with residual sample collected in a vial. The results are reported in an integrated fashion, so that an abnormality seen in either test determines the final result.

The National Cervical Screening Program in Australia has announced the *Renewal* of the National Cervical Screening Program. The aim is to “review the science and technologies related to the program to ensure that all Australian women have access to a cervical screening program that is based on the best available evidence and promotes best clinical practice” [11]. A new evaluation of LBC will be considered as part of this process. In this context, the aim of the current study was to provide accurate data on prevalence of adjunctive LBC use, and the important factors influencing use.

## Methods

We obtained unit record data from the population-based NSW Pap Test Register (PTR) [12] for all women 20–69 years of age who had cervical cytology performed in NSW in fiscal years (FY) 2006/7 to 2009/10. The PTR holds and links individual test records for each identifiable woman. All pathology laboratories in NSW have provided details to the PTR of cervical cytology or pathology tests performed since 1996. Since 2006, cervical cytology tests have been classified as having been read from conventional cytology alone or from conventional cytology with adjunctive LBC. A very small proportion of tests (0.23%) were reported as using LBC alone, and these were excluded from the current analysis. A small proportion of women (0.8%) choose not to have identifiable records kept by the PTR [13], and these were also excluded from the analysis, because test records from these women could not be linked to each other to obtain longitudinal information.

To assess the prevalence of LBC uptake, we used the first cervical cytology test for each woman who had cytology in each quarter, and calculated the age-standardised proportions of these tests for which adjunctive LBC was used, using the Australian 2001 standard population. We used linear regression to examine trends in LBC uptake over the period of interest. Because it is possible that women with a prior abnormality might be more likely to elect to have adjunctive LBC testing, we also assessed prevalence of use according to whether or not the woman had a prior abnormal cytology or histology test, including cytological predictions of possible low grade squamous intraepithelial lesions (pLSIL; broadly equivalent to ASCUS in the Australian Modified Bethesda System of cytological classification) in the past 5 years. We did not include in the analysis a small number of tests (3.8%) that were preceded in the previous 5 years by a test with an unsatisfactory result and an otherwise normal test history.

To examine predictors of LBC use, we identified a cohort of women aged 20–67 years (and who were thus in the age group recommended for screening for at least two subsequent years) who had a cervical cytology test (the index test) in the period 1 July 2006 to 30 March 2008, and who had at least one subsequent test within 27 months (the period of 27 months was chosen since in NSW if a woman does not attend for screening at the 2-yearly recommended interval, a reminder letter is sent by the register at 27 months after her last screening test). We excluded any women with a history of cytological or histological abnormalities or an unsatisfactory result in the 5 years before the index test. Logistic regression was used to calculate odds ratios (ORs) for use of LBC in the subsequent test, adjusted for age and then fully adjusted for all other potential predictors of LBC use in the analysis. The potential predictors included the type of index test (conventional cytology or adjunctive LBC), the index test result (normal, abnormal or unsatisfactory), the type of provider, allocated with reference to discipline code (general practitioner, gynaecologist or other/unknown provider), area of residence (major city, inner regional area, outer regional area, remote/very remote) [14] and socioeconomic status (in quintiles of the index of relative socioeconomic disadvantage based on characteristics of the local government area of residence as ascertained at the 2006 Australian Census) [15].

Human research ethics committee approval for the study was obtained from the NSW Population and Health Services Human Research Ethics Committee. All authors had full access to the de-identified dataset used for analysis (including interim statistical reports and tables).

## Results

### Prevalence of adjunctive LBC use

**Figure 1:**
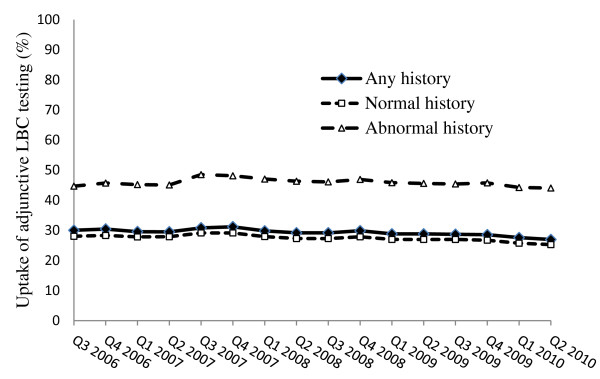
Adjunctive LBC uptake in NSW women,* according to screening history in previous 5 years: FY2006-2010. *Using the first cervical cytology test for each woman who had cytology in each quarter, and calculating the age-standardised proportions of these tests for which adjunctive LBC was used, using the Australian 2001 standard population (see text for more detail).

Figure 1 shows the age-adjusted percentage uptake of adjunctive LBC over the 4 year period from FY2006/7 to FY2009/10, and Additional file 1: Table S1 shows the numbers of women being screened per quarter and the proportion choosing to have LBC. Uptake fell slightly from 29.7% (95% confidence interval (CI) 29.5%-30.0%) in the third quarter (Q3) of 2006 to 26.6% (95% CI 26.4%-26.9%) in Q2 of 2010 (test for trend in decline; p < 0.001). In women with entirely normal cervical cytology in the preceding 5 years, LBC uptake fell slightly from 28% (95% CI27.7%-28.3%) in Q3 of 2006 to 25.3% (95% CI:25.0%-25.5%) in Q2 of 2010 (test for trend in decline; p < 0.001). In women with one or more abnormal cervical cytology results in the preceding five years, LBC uptake remained stable over the period: 44.7% (95% CI:43.9%-45.4%) in Q3 of 2006 and 44.0% (95% CI:43.1%-44.9%) in Q2 of 2010.

### Predictors of adjunctive LBC use

In women who had neither an abnormal nor an unsatisfactory cytology test in the 5 years before their index cytology test, age, place of residence and socioeconomic status were each independent and significant predictors of adjunctive LBC use in the subsequent test (Table 1). The ORs for LBC use rose with increasing age to a peak at 30–44 years then fell again. Relative to an OR of 1 at 35–39 years of age, an estimated 20% reduction in the odds of having adjunctive LBC was observed in women aged 20–24 years and 60+ years of age. Adjunctive use of LBC for the index cervical cytology was by far the strongest predictor of LBC use in a subsequent cervical cytology test; when LBC was used in the index cytology, the odds of using LBC in the subsequent test were increased by more than 13 times, even after adjusting for all other factors (OR 13.6, 95% CI:13.3-13.8). An abnormal result or an unsatisfactory result in the index cytology test also strongly predicted LBC use in the subsequent test, more than doubling the odds of using LBC in the subsequent test (OR 2.62, 95% CI:2.53-2.72) for abnormal and OR 2.37, 95% CI:2.27-3.47 for unsatisfactory tests, respectively). Gynaecologists were more likely than GPs and other providers to request LBC (OR 1.50, 95% CI:1.46-1.54), even after taking into account other factors. Uptake was highest in women in major cities and of highest socioeconomic status (reference ORs of 1) and least for women in remote/very remote areas (OR0.68, 95% CI:0.57-0.80) or in the lowest SES quintile (OR 0.41, 95% CI: 0.40-0.42).

**Table 1:** Odds ratios for predictors of adjunctive LBC uptake in NSW

	**Number of women**	**LBC use %**	**Age-adjusted OR**	**95% CI**	Fully adjusted OR*	**95% CI**
*Age (years)*						
20-24	19,099	27.8	0.74	0.71-0.77	0.82	0.78-0.86
25-29	30,062	31.4	0.88	0.85-0.91	0.92	0.89-0.96
30-34	42,628	34.1	0.99	0.97-1.02	0.98	0.95-1.02
35-39	49,313	34.2	1	-	1	-
40-44	49,019	33.8	0.98	0.95-1.01	1.02	0.99-1.05
45-49	49,318	31.5	0.88	0.86-0.91	0.94	0.91-0.97
50-54	43,109	29.6	0.81	0.79-0.83	0.90	0.86-0.93
55-59	38,589	27.8	0.74	0.72-0.76	0.84	0.81-0.87
60+	38,336	25.2	0.65	0.63-0.67	0.79	0.76-0.82
*P-value*^*‡*^			*<0.001*		*<0.001*	
*Area of residence*						
Major city	196,046	37.2	1	-	1	-
Inner regional	121,486	26.6	0.62	0.61-0.63	0.83	0.81-0.85
Outer regional	38,702	14.3	0.29	0.28-0.30	0.66	0.63-0.68
Remote or very remote	2,362	11.6	0.22	0.20-0.25	0.68	0.57-0.80
*P-value*^*‡*^			*<0.001*		*<0.001*	
*Socioeconomic status*						
1st quintile (highest)	96,000	50.3	1	-	1	-
2nd quintile	68,520	35.4	0.54	0.53-0.55	0.68	0.66-0.70
3rd quintile	71,798	20.5	0.26	0.25-0.26	0.44	0.43-0.46
4th quintile	61,803	20.9	0.26	0.26-0.27	0.45	0.43-0.46
5th quintile (lowest)	60,475	17.9	0.22	0.21-0.22	0.41	0.40-0.42
*P-value for trend*^*‡‡*^			<0.001		<0.001	
*Use of adjunctive LBC in index cytology*						
Conventional cytology only	249,037	13.9	1	-	1	-
Adjunctive LBC	106,819	70.9	15.13	14.87-15.40	13.58	13.33-13.84
*P-value*^*‡*^			*<0.001*		*<0.001*	
*Result of index cervical cytology*						
Normal	324,432	29.8	1	-	1	-
Abnormal	21,009	49.9	2.42	2.35-2.49	2.62	2.53-2.72
Unsatisfactory	13,996	30.3	1.03	0.99-1.06	2.37	2.27-2.47
*P-value*^*‡*^			*<0.001*		*<0.001*	
*Provider type for subsequent cytology*						
General practitioners	293,325	29.3	1	-	1	-
Gynaecologists	49,327	44.6	1.91	1.89-1.95	1.50	1.46-1.54
Other and unknown	2,764	21.0	0.65	0.59-0.71	0.73	0.65-0.82
*P-value*^*‡*^			*<0.001*		*<0.001*	

*OR adjusted for age, index smear cytology result, index smear type of test (conventional cytology or conventional cytology with adjunctive LBC), SES, provider type, and area of residence.

^*‡*^ Obtained from global multivariable model.

^*‡‡*^Obtained from global multivariable model with test for trend performed.

Predictors of adjunctive uptake in a cohort of 360,247 women aged 20-67 years, attending for cervical screening for an index cervical cytology in the period 1 July 2006 to 30 March 2008.

We also assessed evidence for interaction between the variables by including terms for multiplicative interaction in the fully adjusted regression models. We identified some evidence for interaction between socioeconomic status and age in predicting LBC uptake. Therefore, in order to more fully describe LBC uptake in relation to these variables we performed more detailed analysis as follows: (1) We created a compound variable based on broad categories of age (<40; 40+ years) and socioeconomic status (highest three quintiles; lowest two quintiles). Compared to women <40 years in the higher SES category, the fully adjusted odds of adjunctive LBC uptake in women of this age group in the lower SES category were 0.65 (95% CI:0.64-0.67); the odds in women 40+ years in the higher SES category were 0.98 (CI:0.95-0.98) and the odds in women in this age group in the lower SES category were 0.62(CI:0.60-0.63). (2) Using 5-yearly stratification by age, but constructing separate models, we found that the odds ratios for LBC uptake for lower vs. higher SES were 0.63(CI:0.61-0.65) and 0.65(0.63-0.67) for women <40, 40+ years, respectively; and the result in each age group by quintiles of SES were very similar to our main findings for women of all ages. Results were also similar if broad age classifications were made according <30,30+ years and <50,50+ years. Therefore, though the findings were very similar across both age groups for the effect of SES, a slight interaction was noted with age such that the odds of LBC uptake in lower SES women at older ages were slightly higher than for lower SES women at younger ages.

## Discussion

To our knowledge, this is the first study that provides information on prevalence and predictors of use of liquid-based cytology (LBC) for cervical screening in an Australian setting. We used data from a state-based registry in which only a small proportion of women (<1%) chose not to disclose identifiable information. The study was also conducted in the context of a relatively high overall screening participation rate in NSW, in which 58.8% of women aged 20–69 years were screened every 2 years [13] and 71.2% every 3 years in 2007-8 [13]. We found that adjunctive uptake of LBC in NSW is high, at approximately 27-30%, but that the decision to use LBC depends strongly on a woman’s age and previous screening history, and use of the technology is increased in high socioeconomic status and urban groups. Therefore, the study demonstrates that uptake of LBC, to date, has not been comparable for all groups of women; these findings are relevant to considerations of equity when new technologies for cervical screening are made available.

It is notable that the observed pattern of LBC uptake with age, which peaks in women in their thirties and forties, to an extent mirrors patterns in overall screening participation which peaks in women in their thirties, forties and fifties [9,13]. Screening participation has also been shown to depend on socioeconomic status and area of residence [9,13]. Taken together with the current study, these findings suggest that some of the factors that influence women to participate in cervical screening might also be involved in the decision to use LBC. Such factors could be related to financial issues, access to screening services, or understanding of the benefits of cervical screening. We also found that use of LBC was more likely when the test provider was a specialist gynaecologist, even in a group of women selected to have a normal history for 5 years prior to the index test, and after adjusting for having an abnormal index smear (which may have prompted the specialist referral). It is possible that greater awareness about LBC technology amongst specialist providers is a factor in this finding; and it is possible that the medical practitioner’s recommendation is a factor in the woman's decision to use LBC. Overall, our findings appear broadly comparable to historical findings from the USA where the decision to use LBC as a direct replacement to cytology might have involved additional costs to the woman; in that country it has been found that women with more education or who were living in metropolitan areas were more likely to have LBC testing [16,17].

Because cytology registers are configured at the state level, we were not able to analyse national data in the current study. The rates of LBC use in NSW will have a considerable influence on national rates since the population in NSW comprises approximately one third of the Australian population. However, our findings for prevalence of uptake are not likely to be generalisable to other states – for example, uptake in Victoria in 2011 was 4% overall (personal communication, A/Prof. Marion Saville), whereas to our knowledge, detailed information on levels of uptake in other states have not been reported. Probable differences in the overall levels of LBC uptake between states probably depend on a range of factors including availability of the technology through specific laboratories. Nevertheless, even though absolute rates of uptake in other states probably differ from that in NSW, our finding that certain factors seem to predispose women towards choosing LBC may have broader applicability.

It is important that our findings are set in context of the scale of the potential benefits to women of having LBC testing. The test sensitivities for conventional cytology and manually-read LBC for detection of biopsy-confirmed CIN2+ lesions are similar [3,4], and there is no evidence, to our knowledge, to suggest that adjunctive testing *per se*, improves overall sensitivity. However, there is some initial evidence from two Australian studies that image-read LBC may deliver greater benefits in terms of test sensitivity [5,6]. Although in this study we were unable to quantify the proportion of adjunctive tests in NSW involving image analysis of the slide (because this information is not recorded in the NSW PTR), it is likely that a very high proportion of tests since 2008 have involved imaging, because the two laboratories processing the largest volumes of cytology slides now routinely use the technology (personal communication. Prof. Annabelle Farnsworth).

Modelled analysis has previously predicted that under favourable assumptions about LBC test accuracy, if image-read LBC were to be introduced as a direct replacement to conventional cytology in Australia, 68 additional cervical cancer cases and 19 deaths would be prevented, but 26 additional treatments for high grade precancerous lesions would be required for each case prevented [4]. However, this balance of benefits and harms also depends on other factors such as the age range for screening and the screening interval, and is also likely to change over time due to the effects of HPV vaccination. The National HPV Vaccination Program, introduced in 2007, involves routine vaccination of 12–13 years old girls and a catch-up of girls and women aged 12–26 years which was conducted to 2009; there are already initial indications of a potentially vaccine-related drop in biopsy-confirmed CIN2+ rates in Australian females less than 18 years of age [18]. These changes are likely to have a rapid effect on the balance of benefits and harms (and cost-effectiveness) of using LBC technology.

The findings of our study are timely. They provide important context on current patterns of uptake of LBC, which is an important candidate technology for inclusion in the National Cervical Screening Program. The scope of the Renewal process also includes evaluation of HPV DNA testing both as a triage test for women with low grade cytological abnormalities, and as a primary screening test. Consideration of LBC as a direct replacement for conventional cytology within the national program will likely take into account the potential to perform HPV testing from the LBC sample, as well as the impact of screening interval and age range on the balance of benefits and harms associated with a move to LBC. This integrated evaluation of changes to screening technology, in context of other aspects of screening, seems warranted.

## Conclusions

In the environment in Australia in which public funding for LBC has not been available, adjunctive uptake of LBC depends strongly on a woman’s age, her screening history and socioeconomic factors. These findings provide important context for a current review of technologies used in the National Cervical Screening Program in Australia.

## Abbreviations

LBC: Liquid based cytology; HPV: Human papillomavirus; MSAC: Medical Services Advisory Committee; Pap: Papanicolaou smear; PTR: NSW Pap test register; ORs: Odds ratios.

## Competing interests

Karen Canfell receives salary support from the National Health and Medical Research Council, Australia. She is co-PI of a new trial of primary HPV screening in Australia, for which the pilot study is partially supported by Roche Medical Systems and Ventana Medical Systems, USA.

## Authors’ contributions

All authors were involved in design of the protocol and preparation of the Human Research Ethics Committee application. NA was responsible for data analysis and contributed to drafting the manuscript. BA and KC supervised and supported data analysis, performed additional analysis and drafted the manuscript. All authors read and approved the final manuscript.

## Pre-publication history

The pre-publication history for this paper can be accessed here:

http://www.biomedcentral.com/1471-2458/13/1196/prepub

## Supplementary Material

Additional file 1: Table S1 LBC uptake over the period 2006-2010, in women aged 20-69 years, by smear history over the previous 5 years. Click here for file
