# Supplementary material for: Uptake of liquid-based cytology as an adjunct to conventional cytology for cervical screening in NSW, Australia: a cross-sectional and population-based cohort analysis
Source: BMC Public Health. 2013 Dec 18;13:1196. doi: 10.1186/1471-2458-13-1196 (PMC3890550; doi:10.1186/1471-2458-13-1196)
Supplement: Additional file 1: Table S1 — LBC uptake over the period 2006-2010, in women aged 20-69 years, by smear history over the previous 5 years. [file 1471-2458-13-1196-S1.docx]

**Supplementary Table 1: LBC uptake over the period 2006-2010, in women aged 20-69 years, by smear history over the previous 5 years.**

| Quarter/year | Any history | | Normal history | | Abnormal history | |
| --- | --- | --- | --- | --- | --- | --- |
|  | Total (N) | LBC use (%) | Total (N) | LBC use (%) | Total (N) | LBC use (%) |
| Q3 2006 | 152065 | 30.1 | 98234 | 28.7 | 21143 | 45.2 |
| Q4 2006 | 153123 | 30.5 | 98603 | 28.9 | 20646 | 46.7 |
| Q1 2007 | 165849 | 29.6 | 106637 | 28.5 | 20128 | 45.9 |
| Q2 2007 | 174713 | 29.5 | 112469 | 28.5 | 19853 | 46.1 |
| Q3 2007 | 168174 | 30.8 | 106787 | 29.8 | 19733 | 49.0 |
| Q4 2007 | 155836 | 31.1 | 100044 | 29.9 | 19435 | 48.9 |
| Q1 2008 | 167695 | 29.8 | 108013 | 28.6 | 20451 | 47.5 |
| Q2 2008 | 174905 | 29.2 | 114400 | 28.0 | 20614 | 47.0 |
| Q3 2008 | 160612 | 28.8 | 105116 | 28.0 | 19390 | 46.9 |
| Q4 2008 | 147713 | 29.8 | 96973 | 28.5 | 18564 | 47.5 |
| Q1 2009 | 171978 | 28.8 | 114313 | 27.7 | 20101 | 46.6 |
| Q2 2009 | 171978 | 28.8 | 114426 | 27.7 | 20316 | 46.3 |
| Q3 2009 | 157356 | 28.7 | 106815 | 27.6 | 17818 | 46.0 |
| Q4 2009 | 143044 | 28.5 | 97695 | 27.3 | 16515 | 46.2 |
| Q1 2010 | 161872 | 27.5 | 110520 | 26.4 | 18146 | 44.7 |
| Q2 2010 | 158895 | 26.9 | 108796 | 25.9 | 17080 | 45.0 |
